# Fast and Sensitive Interferon-γ Assay Using Supercritical Angle Fluorescence

**DOI:** 10.3390/bios3010108

**Published:** 2013-02-08

**Authors:** Christian M. Winterflood, Thomas Ruckstuhl, Stefan Seeger

**Affiliations:** Physikalisch-Chemisches Institut, Universität Zürich, Winterthurerstrasse 190, CH-8057 Zürich, Switzerland; E-Mails: c.winterflood@pci.uzh.ch (C.M.W.); t.ruckstuhl@pci.uzh.ch (T.R.)

**Keywords:** one-step immunoassay, interferon-gamma, supercritical angle fluorescence, polymer test tube

## Abstract

We present an immunoassay for Interferon-γ (IFN-γ) with a limit of detection of 1.9 pM (30 pg/mL) and a linear concentration range spanning three orders of magnitude. The developed one-step assay takes only 12 min and can replace the time-consuming and labor-intensive enzyme-linked immunosorbent assay (ELISA). The solid-phase sandwich assay is performed on a new measurement system comprising single-use test tubes and a compact fluorescence reader. The polymer tubes contain an optical configuration for the detection of supercritical angle fluorescence, allowing for highly sensitive real-time binding measurements.

## 1. Introduction

Interferon-γ (IFN-γ) is a small, homodimeric protein mainly produced by T-cells and natural killer cells. It plays key functions in host defense against pathogens by exerting anti-viral, anti-proliferative and immunoregulatory activities. Its sensitive and accurate quantification is therefore relevant in medical research and diagnostics. The most widely used method for the quantification of bioanalytes is ELISA. A typical ELISA protocol involves dozens of washing and incubation steps, takes several hours and requires relatively large amounts of expensive antibody conjugates. The need for better solutions is driving the development of new assay technologies using magnetic micro- and nano-particle [1,2,3,4,5] surface plasmon resonance [6,7], surface enhanced Raman scattering [8], electrical signals [9], electro-chemiluminescence [10,11] and fluorescence [12,13]. 

**Figure 1:**
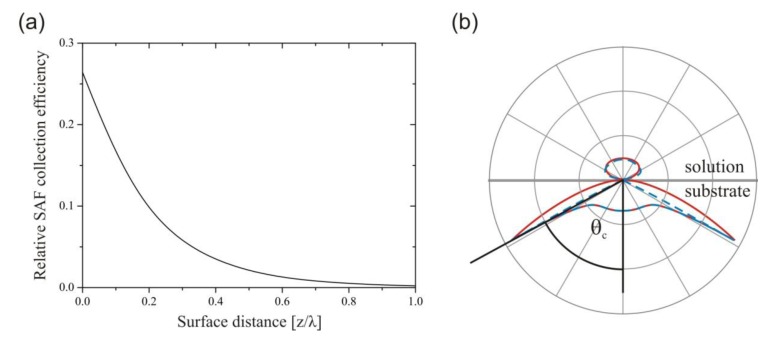
Supercritical angle fluorescence (SAF) emission at the solution/substrate interface. (**a**) The relative SAF collection efficiency of the parabolic element with increasing surface distance *z*, expressed as a fraction of the emission wavelength, λ. (**b**) Polar plots of the angular emission distribution of a fluorophore with distances *z *= 0 (red, solid) and *z *= λ/3 (blue, dashed) from the interface. Adapted from [14].

We present a one-step sandwich immunoassay for the quantification of recombinant mouse IFN-γ (rmIFN-γ) within minutes. The assay has been developed on the supercritical angle fluorescence (SAF) immunodiagnostic system, a recently reported fluorescence-based detection platform comprising single-use test tubes and a compact fluorescence reader [14]. The mass producible polymer tubes contain an innovative optical configuration for the collection of SAF, which is emitted above the critical angle into the solid substrate. As shown in Figure 1, SAF occurs only for fluorescent molecules located near the surface (<200 nm). This property of SAF finds application in high resolution microscopy [15,16] or the monitoring of binding reactions in real-time at surfaces [17,18,19]. 

In a sandwich-assay format, as presented here, the collection of SAF allowed for detection of the formation of sandwich complexes at the surface, with little contribution from fluorescent detection of free antibody in solution. Picomolar rmIFN-γ concentrations were measured effortlessly within 12 min without the need for any washing steps.

## 2. Experimental Section

### 2.1. SAF Immunodiagnostic System

**Figure 2:**
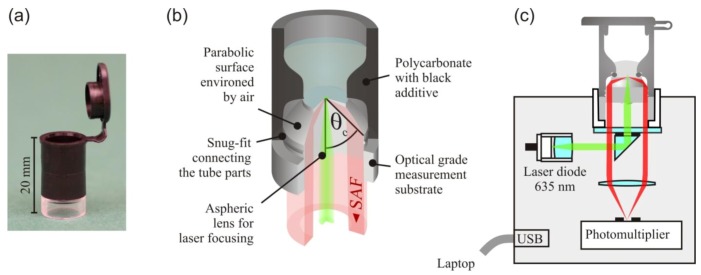
(**a**) Photograph and (**b**) schematic of the test tube. (**c**) Schematic of the fluorescence reader. Adapted from [14].

The SAF immunodiagnostic system, shown in Figure 2, comprises mass producible polymer test tubes and a compact fluorescence reader. The tube consists of two polymer components and a standard O-ring. The lower part containing the optics was fabricated by injection molding of the cyclo-olefin polymer Zeonex™ (Zeon Chemicals, Tokyo, Japan). An aspheric surface at its bottom side focuses collimated excitation light to a light disk of 50 μm diameter at the upper surface of the substrate. The excited fluorescence bound at the substrate is collected at angles between 63° and 78° by a parabolic shell surface environed by air. The collected angles lie above the critical angle between the aqueous sample and the substrate of θ_c_ = 61°. The fluorescence collection efficiency of the tube optics is with ~27%, comparable to the collection efficiency of the microscope objectives of high numerical aperture. The cylindrical base of the substrate is the connector between the tube and fluorescence reader. The optical interfaces (asphere, bottom flat) are concealed inside this hollow cylinder to prevent damage and contamination. The substrates were fabricated with high optical accuracy by Syntec Optics (Pavillion, NY, USA). After the immobilization of capture antibodies on the upper flat surface, the substrate was assembled, with the upper part of the tube injection molded from polycarbonate and black additive. A snug fit establishes a strong junction between the tube parts. The tube has the same diameter (10.7 mm) as its well-known counterpart from Eppendorf and is therefore compatible with common laboratory equipment (shakers, centrifuges, thermocyclers, *etc*.). 

The fluorescence reader is a portable device operated via USB from a laptop. For fluorescence excitation, a 635 nm diode laser is used. A neutral density filter on a motorized filter wheel is used to switch between the excitation intensities of 1 μW and 1 mW. A small reflection prism separates the optical paths of fluorescence excitation and detection. A photomultiplier unit is used to detect the fluorescence. For the measurement, the tube is inserted into a cylindrical cavity on the top of the device.

### 2.2. Preparation of Capture Antibodies and Detection Antibodies

Antibody immobilization on Zeonex™ was done, as described in [20]. In brief, the Zeonex substrates were activated by oxygen plasma (40 kHz/100 W/0.2–1 mbar) on a Femto plasma device (Diener Electronic, Ebhausen, Germany) for 5 min and silanized by immersion in a 3% (v/v) solution of 3-aminopropyl triethoxysilane (Sigma-Aldrich, St. Louis, MO, USA) in ethanol for 2 h. The tubes were rinsed with ethanol and water, dried under nitrogen flow and left to cure overnight. The silanized Zeonex™ was functionalized with aldehyde-activated dextran by Schiff’s base coupling. For this, it was immersed in a solution of 2% (w/v) dextran T40 (Carl-Roth, Karlsruhe, Germany) and 30 mM sodium periodate (Sigma-Aldrich) for 2 h, rinsed with double-distilled H_2_O (ddH_2_O) and further oxidized in 30 mM sodium periodate for 2 h. The Zeonex™ substrates were assembled with the O-ring and the upper tube part. Streptavidin (Sigma-Aldrich) was immobilized by Schiff’s base coupling by filling the tubes with 50 μL of a 1 mg/mL solution of phosphate buffered saline (0.01 M PBS, pH 7.4) and incubating over night at 4 °C. The tubes were further incubated with 100 μL of 5 mM glycine/PBS to block unreacted aldehydes. The tubes were washed several times with 0.05% (v/v) Tween 20 in PBS. For later use, the tubes were incubated with 100 μL of Liquid Plate Sealer (Candor Bioscience, Wangen, Germany) for 1 h at 4 °C. The solution was removed, and the tubes were dried under nitrogen flow. After this treatment, the tubes can be stored at 4 °C in dry conditions for a longer period. For the presented experiments, the storage time was within 2 weeks. Prior to the assays, the tubes were incubated for 1 h with 50 μL of biotinylated capture antibody (rat-anti mouse IFN-γ clone R4-6A2, eBioscience, San Diego, CA, USA) at 30 μg/mL in PBS and washed several times with 0.05% (v/v) Tween 20 in PBS. This capture antibody concentration was shown to completely saturate the available streptavidin binding sites and was large enough to reach saturation within 1 h. The tubes were blocked for 1 h with 3% (w/v) bovine serum albumin/0.05% (v/v) Tween 20 in PBS. The assays were performed using recombinant mouse IFN-γ (Invitrogen, Carlsbad, CA, USA) in 3% (w/v) bovine serum albumin/0.05% (v/v) Tween 20 in PBS. The detection antibody (rat-anti mouse IFN-γ clone AN-18, eBioscience) was labeled with the red fluorescent dye, Cy5 (Invitrogen), using standard N-hydroxysuccinimidyl coupling chemistry, yielding a dye-to-protein ration of ~1.7. To photobleach the autofluorescence of the substrates, the tubes were irradiated with a 635 nm high brightness LED for 1 h.

### 2.3. Assay Procedure

The rmIFN-γ concentration measurements were carried out in a sandwich test format using two monoclonal antibodies with orthogonal site specificity. The binding of the analyte molecules to the capture antibodies and the detection antibodies was performed in one step, and the formation of the resulting sandwich complexes was monitored in real-time. The short assay protocol reads as follows:

(1) Pipette 5 μL 100 nM detection antibody solution into tube;
(2) Pipette 45 μL rmIFN-γ into tube;
(3) Insert the tube into the reader instrument and start measurement.

During the first 700 s, the binding was monitored by 1 s integration of the SAF intensity using a low excitation intensity of 1 μW. The sampling interval was increased by 1 s after each sampling. For samples with low signals, a sensitive readout mode was carried out automatically after 700 s using a high excitation intensity of 1 mW, and the SAF was then collected every 2 s with 1 s integration time. Thereby, the fluorophores were photobleached, and the amplitude of the intensity decay served as a sensitive measure for the amount of rmIFN-γ bound at the surface [21,22].

## 3. Results and Discussion

**Figure 3:**
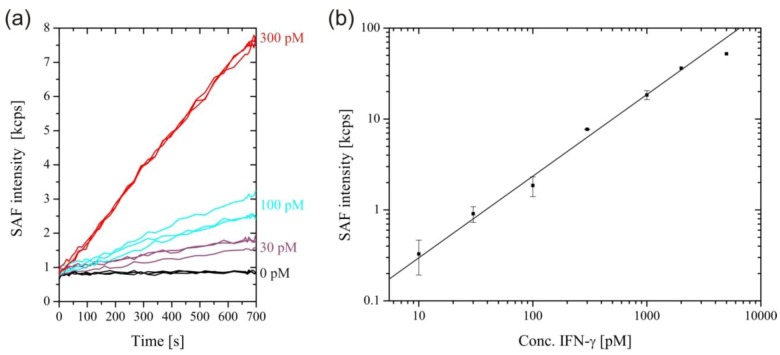
(**a**) Real-time measurement of selected concentrations of Interferon-γ (IFN-γ) . (**b**) SAF intensity increase after 700 s plotted against IFN-γ concentration. A straight line through the origin was fitted to the data for IFN-γ concentrations up to 2 nM (Adjusted R-squared = 0.995). The linear relationship is lost at higher concentrations due to depletion of the free detection antibody.

Figure 3(A) shows SAF intensity curves for the measurement of selected rmIFN-γ concentrations. Due to the excellent sensitivity of the system, fairly smooth binding curves were obtained for picomolar analyte concentrations. The binding followed a rather complex kinetics, as the sandwich formation at the surface proceeded through two pathways, with rmIFN-γ molecules binding to either the detection antibody in solution or to the capture antibody on the surface first. The calibration curve shown in Figure 3(B) was obtained by plotting the rmIFN-γ concentrations from 10 pM to 5 nM *versus* the SAF intensity measured after 700 s. The saturation of the signal for rmIFN-γ concentrations above 2 nM was caused by the depletion of the detection antibodies. 

**Figure 4:**
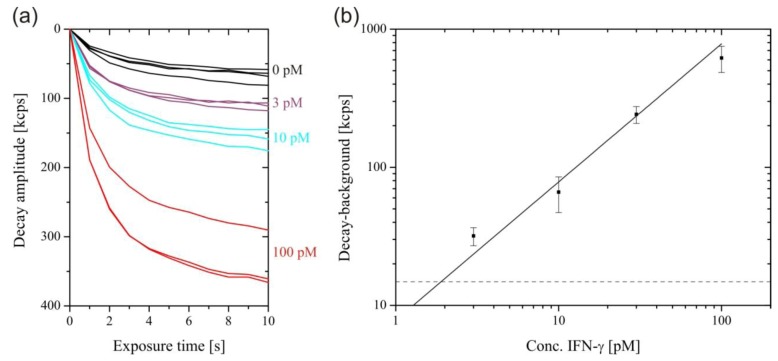
Sensitive readout after 700 s with 1 mW excitation. (**a**) Photobleaching decays of the SAF intensity. (**b**) Plot of the photobleaching amplitudes after 11 s, minus the background (zero concentration decay) against IFN-γ concentration. A straight line through the origin was fitted through the data points (Adjusted R-squared = 0.980). The dashed line corresponds to the 3σ value of the zero concentration measurement.

For the measurement of low rmIFN-γ concentrations, the excitation intensity was increased to 1 mW after 700 s, enhancing the SAF intensity by three orders of magnitude and causing the surface-bound fluorophores to photobleach within a few seconds. Figure 4(A) shows the intensity decays during photobleaching for selected rmIFN-γ concentrations. The intensity decay obtained in the absence of rmIFN-γ was caused by non-specific adsorption of detection antibodies at the surface. This background was subtracted from the rmIFN-γ concentration-dependent decay amplitudes. The plot of the background-corrected decay amplitude *versus* rmIFN-γ concentration is shown in Figure 4(B). The data were fitted by a straight line through the origin, and the limit of detection was calculated by its intersection with three-times the standard deviation (3σ value) of the zero concentration measurements, to 1.9 pM. Accordingly, the concentration range of the measurement was 30–32,000 pg/mL, with an assay time of only 12 min. For comparison, the supplier of the employed antibodies (eBioscience) specifies the recombinant standard range for the ELISA of 15–2,000 pg/mL, with an assay time of 4 h. In the one-step sandwich assay performed with the SAF immunodiagnostic system, there is a trade-off between sensitivity and dynamic range. The detection of high rmIFN-γ concentration requires the use of high detection antibody concentrations, leading to an elevated background. The use of a lower detection antibody concentration shifts the dynamic range towards even lower rmIFN-γ concentrations. The mean coefficient of variation of 14.8% of the assay was larger compared to commercial ELISA kits, which is around 10%. This comparably large variation can mainly be ascribed to variations in the capture antibody density from tube to tube, as a result of the manual immobilization procedure.

## 4. Conclusions

We have developed a rapid and sensitive assay for IFN-γ on the SAF immunoassay platform. The assay is about twenty-times faster than standard ELISAs for IFN-γ and has a comparable linear concentration range. A comparison between the SAF assay and several commercially available ELISA kits for IFN-γ is given in Table 1. The linear measurement range of the SAF assay can be shifted towards lower concentrations straightforwardly by using a lower concentration of detection antibody. The SAF assay scheme is extremely economical regarding the material requirements. The assay requires fewer substances, and the required amounts of expensive detection antibody and capture antibody is only a fraction of what is needed for an ELISA. The amount of antibody required for the SAF assay can be further reduced by confining the immobilization of capture antibodies to the detection region on the substrate. The portable SAF immunoassay platform combines high detection performance with low cost and brings sensitive testing to where it is required. It addresses the need for fast and effortless concentration measurements and should replace the time-consuming and laborious ELISA. 

**Table 1:** Comparison between the SAF assay and the specifications of several commercially available ELISA kits for mouse IFN-γ.

*Assay*	*Linear range*	*Time requirement*
eBioscience, Inc.	15–2,000 pg/mL	4½ h
Thermo Fischer Scientific, Inc.	37–3,000 pg/mL	4 h
Abcamm, Inc.	31–1,000 pg/mL	3¾ h
BioLegend, Inc.	30–2,000 pg/mL	4 h
Cisbio, Inc.	7.8–2,000 pg/mL	2 h
**SAF assay**	**30**–**32,000 pg/mL**	**12 min**
